# Birds of a feather flock together: Insights into starling murmuration behaviour revealed using citizen science

**DOI:** 10.1371/journal.pone.0179277

**Published:** 2017-06-19

**Authors:** Anne E. Goodenough, Natasha Little, William S. Carpenter, Adam G. Hart

**Affiliations:** 1 School of Natural & Social Sciences, Francis Close Hall, University of Gloucestershire, Cheltenham, Gloucestershire, United Kingdom; 2 Royal Society of Biology, Charles Darwin House, London, United Kingdom; Rijksuniversiteit Groningen, NETHERLANDS

## Abstract

Pre-roost murmuration displays by European starlings *Sturnus vulgaris* are a spectacular example of collective animal behaviour. To date, empirical research has focussed largely on flock movement and biomechanics whereas research on possible causal mechanisms that affect flock size and murmuration duration has been limited and restricted to a small number of sites. Possible explanations for this behaviour include reducing predation through the dilution, detection or predator confusion effects (the “safer together” hypotheses) or recruiting more birds to create larger (warmer) roosts (the “warmer together” hypothesis). We collected data on size, duration, habitat, temperature and predators from >3,000 murmurations using citizen science. Sightings were submitted from 23 countries but UK records predominated. Murmurations occurred across a range of habitats but there was no association between habitat and size/duration. Size increased significantly from October to early February, followed by a decrease until the end of the season in March (overall mean 30,082 birds; maximum 750,000 birds). Mean duration was 26 minutes (± 44 seconds SEM). Displays were longest at the start/end of the season, probably due to a significant positive relationship with day length. Birds of prey were recorded at 29.6% of murmurations. The presence of predators including harrier *Circus*, peregrine *Falco peregrinus*, and sparrowhawk *Accipiter nisus* was positively correlated with murmuration size (R^2^ = 0.401) and duration (R^2^ = 0.258), especially when these species were flying near to, or actively engaging with, starlings. Temperature was negatively correlated with duration but the effect was much weaker than that of day length. When predators were present, murmurations were statistically more likely to end with all birds going down *en masse* to roost rather than dispersing from the site. Our findings suggest that starling murmurations are primarily an anti-predator adaptation rather than being undertaken to attract larger numbers of individuals to increase roost warmth.

## Introduction

Collective animal behaviour, where multiple individuals of a species act in a highly-coordinated manner, is both taxonomically and ecologically widespread [1]. Examples include fish schooling to distract predators (e.g. banded killifish *Fundulus diaphanous*) [2], ants collectively foraging using pheromone trails [3], and herds of migrating blue wildebeest *Connochaetes taurinus* [4]. Many collective behaviours exhibit characteristics of self-organisation, whereby relatively simple repeated interactions produce complex emergent patterns [5–7]. In many cases, environmental factors modulate individual interactions, such that different collective behaviours emerge under different conditions [8]. Collective behaviours are assumed to be adaptive [1] and understanding the mechanisms by which they emerge is a key area of research.

One spectacular example of collective behaviour is the pre-roost aerial displays undertaken by European starlings *Sturnus vulgaris* [9–10]. These murmurations (so-called because of the sound produced by multiple wingbeats) can involve thousands of individual birds forming a coherent three-dimensional murmuration “cloud” within which the movement of each individual bird is highly cohesive and synchronized. This synchronised movement means that the group forms a range of different shapes [11–12] including spheres, planes and waves [13–14] while remaining more-or-less static with respect to a focal point on the ground (generally the roosting site). Typically occurring at sunset, murmurations generally end with the birds descending *en masse* to roost.

As highlighted by King and Sumpter [10], murmuration behaviour is of considerable interest not only to biologists, but also to physicists, engineers and mathematicians. To date, attention has tended to focus on the mechanisms of murmurations to determine the rules governing individual “agent” behaviour and understanding how these rules lead to collective behaviour [15–16]. Much of this work has used computer analysis of rapid-burst photographs or video footage of murmuration events [11–12; 17–18] as well as agent-based simulation modelling [19–22]. For example, photography of murmurations in Rome has allowed insights into the morphology and orientation of starling flocks [11]. Analysis of videos from the same sites has revealed that the distance (correlation length) over which velocity is correlated among neighbours within a murmuration is dependent on flock size, such that the behavioural state of one bird affects, and is affected by, that of all other birds involved in the same event [12]. Tracking individual birds using 3D reconstruction has shown that direction-switching is spatially localised (i.e. the birds that turn first are all co-located within the group) and then propagates through the group through bird-to-bird information transfer [18]. The 3D reconstruction approach has also been used to show that each bird interacts with, and moves according to, six or seven nearest neighbours and it is the proximity of those neighbours, as opposed to all birds within a fixed distance, which dictates individual movement [17].

Somewhat surprisingly given the amount of work on the mechanisms of murmurations, we know little about their adaptive value or what factors affect size and duration [10]. One potential explanation is the “warmer together” hypothesis. As murmurations occur immediately before roosting, and during the late autumn and winter months, it is possible that they act to “advertise” a roost site so the roost becomes warmer as more birds gather (producing an inverse relationship between temperature and murmuration duration/size). This would also allow the possibility of individual birds following more successful or experienced individuals to good feeding areas as the roost disperses. This has been seen both for socially-roosting red-billed quelea and cliff swallows *Hirundo pyrrhonota* [23–24] and could be particularly important in cold weather [25]. The “warmer together” hypothesis has not been tested empirically for starlings, but Davis and Lussenhop [26] showed that small flocks 'funnelled' into progressively larger ones along flight-lines to the roost and concluded that social stimulation from aerial displays was important for creating larger roosts. A similar situation has been found in common bushtits *Psaltriparus minimum* where birds form denser roosts in colder weather [27] and in wrens *Troglodytes troglodytes* where birds vocally advertise communal roosts in cold weather [28].

The second hypothesis (or, more correctly, group of hypotheses) is that murmurations are an anti-predator strategy. Starlings are potential prey for a range of avian predators, especially hawks and falcons. Murmurations could reduce predation risk in one of several ways, which are not mutually exclusive:

1. Dilution effect: as group size (N) increases, the chance of any one individual suffering predation (1/N) decreases, favouring larger groups [29–30]. Individual birds could further decrease their individual risk through a separate (but complementary) strategy of seeking more sheltered locations within the flock, with birds on the edge of the flock continually moving towards the centre within a Hamiltonian “selfish herd” [31–32]. Flocking behaviour in other species has been shown to be consistent with selfish herd dynamics (e.g. sand fiddler crabs *Uca pugilator* [33] and European minnows *Phoxinus phoxinus* in structurally-simple habitats [34]).
2. Detection effect: group anti-predator vigilance increases as the number of individuals in the group increases [35–36]. This remains the case even when each individual in a group contributes less vigilance than a typical solitary individual, as shown for yellow-eyed juncos *Junco phaeonotus* and starlings in small feeding flocks [37–38, respectively]. The detection effect often interacts with the dilution effect, as shown by as shown for captive socially-flocking red-billed quelea *Quelea quelea* [39] and red deer (elk) *Cervus elaphus* [40].
3. Predator confusion effect: most aerial predators hunt by targeting a specific bird within a group. The constant movement within murmurations might reduce a predator’s ability to “lock onto” an individual; an effect known as target degeneracy [20]. This has been seen in silvery minnows *Hybognathus nuchalis*, whose movement within groups of almost eliminated depredation by largemouth bass *Micropterus salmoides* [41]. Group size and density might also be important, with predators finding it harder to isolate individuals in larger, denser groups.

There has been little work to test the “safer together” hypotheses for starling murmurations. The dilution and confusion hypotheses have been supported by agent-based modelling of within-flock movements [32, 42], as well as in a computer game experiment on the ability of a (human) predator to “capture” a target starling in a biologically-realistic model [43]. Empirical studies have shown that predation pressure can result in waves of agitation (dark bends moving away from the predator [14]) as a result of changes in bird orientation [44] and in the shape of the murmuration itself [13], but these studies did not analyse the effect of predators on murmuration size or duration. The studies also only considered one predator species (peregrine falcon *Falco peregrinus*) and were based on data from just two sites.

Obtaining detailed data on multiple murmurations at broad spatiotemporal scales presents a considerable challenge [11]. Here, we use a citizen science approach [45] to harness the efforts of thousands of volunteers to record murmurations. Conducted over two years, and amassing >3,000 records from 23 different counties, the project gathered information on murmuration size and duration in relation to location, season, time of day, and habitat (year one) as well as temperature and predator presence and behaviour (year two). This approach overcame the typical limitations inherent in murmuration research to allow, for the first time, a robust analysis of potential triggers of murmuration behaviour.

## Methods

### Citizen science surveys

The first online survey, promoted through the websites of the Royal Society of Biology and the University of Gloucestershire, was created using *Survey Monkey* and prefaced with information on starling murmurations and photographs of murmuration displays. The survey opened on 17^th^ October 2014 with participants able to document records from the beginning of that month (1 October = day 1). The survey remained open until 31^st^ March 2015, with the final record being submitted on 23^rd^ March 2015 (day 174). This spanned the autumn/winter period when murmurations occur. Participants were asked to log observations of murmurations and provide the following baseline information: date; time; location (as postcode, Ordnance Survey grid reference, latitude/longitude or address); whether the location was urban, rural or suburban; and the habitat over which the birds were murmurating. Participants were asked to estimate murmuration size (number of birds), duration (minutes from the start of observation until the display ended), and what happened at the end of the murmuration—if this was observed—to determine whether birds went down to roost or dispersed from the location. Participants could report multiple murmurations over the survey period, including murmurations occurring at the same location on different nights. Participants were advised before the submission step that by continuing with submission they were providing informed consent for participating in the study and their data being used in subsequent published research.

A second online survey was launched on 3^rd^ November 2015 with participants being submit records from the beginning of the preceding month (1 October = day 1). The survey remained open until 31^st^ March 2016 with the final record being submitted on 27^th^ March 2016 (day 179 due to leap year). Participants were again asked to record murmuration size, duration, and what happened at the end of the event but this time were asked for details of temperature (°C) and the presence of other birds. The species that could be recorded were all potential predators (or species that might, in silhouette, look similar to potential predators): kestrel *Falco tinnuculus*, peregrine *Falco peregrinus*, sparrowhawk *Accipiter nisus*, red kite *Milvus milvus*, buzzard *Buteo buteo*, harrier *Circus* spp., owl (order: Strigiformes); corvids (order: Corvidae), or gulls *Larus* spp. The order of species was randomised for each survey to reduce the risk of under- or over-representation by virtue of order of appearance. There was also opportunity for other species to be listed. The activity of potential predators was recorded from the options of: (1) perching and silent; (2) perching and calling; (3) flying but neither close to, nor engaging with, the starlings; and (4) flying very close to, or actively engaging, with the starlings (active engagement being defined as a bird of prey flying through the starling flock or making a predator strike, whether or not this was successful). Finally, people were asked for very brief details of location (nearest town) and when the murmuration had occurred from the options of “today”, “within the last week” or “within the last month” from a drop-down list. This very basic way of collecting location and date information made the survey quicker and avoided duplication with year one but allowed international records to be identified and excluded from certain analyses (e.g. analysis of predators in cases where the potential predator community differed) and exclusion of records of murmurations from more than one month previously when memory of temperature and predator activity could be unreliable.

### Survey publicity

In both years, there was extensive media promotion and coverage of the study including mentions in most UK national newspapers as well as articles in over 200 UK local and regional newspapers and national magazines. The survey was featured on UK regional and national TV news and countryside-related programmes as well the local BBC radio network. A variety on online news sites, blog posts and Facebook sites were used to promote the survey and via a dedicated Twitter account *@Starling_Survey*. Twitter was also searched daily for people mentioning murmuration displays and those users were sent direct tweets asking them to complete the survey by following an embedded link. The majority (ca. 95%) of Twitter invitations were sent in the first three months of the survey each year; in February and March there were fewer mentions of murmurations on Twitter in general and most of these were from people who had received the invitation in response to previous murmuration tweets. Anecdotal observations did not suggest murmurations that were the subject of tweets–and thus invitations to complete the survey–were exceptional in any way (e.g. particularly large or long in duration). Rather, tweeters frequently mentioned that their observation was the first murmuration they had seen, that they happened to get a good photograph or it, or simply that they had enjoyed a display and wanted to alert others to its existence.

### Standardising data

Data from both years were carefully standardised. Historical records (i.e. people recording a murmuration remembered from >1 month previously) were deleted (n = 40), as were incomplete records (n = 167). Records that were obviously inaccurate or unusable were also discarded. These included records where there were no birds present (n = 2), occasions when murmuration duration was given as zero minutes (n = 8), and reports of individuals feeding in gardens/parkland (n = 67). Where location had been provided as a postcode, address or grid reference, this was translated into latitude and longitude. Records outside the UK were highlighted so that they could be included in spatial analyses but excluded from analyses of predators to avoid location being a confounding factor. In each year, a subset of the data was created, which only contained observations of murmurations of ≥500 birds that descended to roost at the end of the display. These were likely to be true murmurations based on both the size of the flock and the ultimate roosting behaviour, rather than simple flocks of birds moving to a new location. The use of this threshold was based on work in Rome where 448 birds and 428 birds were the minima to required produce a definite murmuration pattern [11, 15].

### Additional data

For the UK subset data, average day length was calculated for the geographical centre of the UK as defined by the Ordnance Survey (Dunsop Bridge, Lancashire, UK, 53.9419°N 2.5369°W) using the Sunrise-Sunset mobile application (version 1.03; Peter Smith, petesmith.co.nz/sunrise-sunset-modern). These data were added for each record based on the day that the murmuration was sighted. Although day length was specific only to the date of the murmuration record and not its location, analysis showed that day length in the UK only varies across the survey period by an average of ca. 10 minutes from the centre of the UK to the north of Scotland or the far south of England.

### Statistical analysis

All analyses were undertaken in R Version 3.3.1 [46], SPSS Version 22 for Windows (IBM), or QGIS Version 2.16 [47] using underlying base maps from Natural Earth that are in the public domain. To analyse spatial patterns, QGIS was used to display location data at both global and UK scales. Pearson correlation was then used to quantify any relationship between: (1) the number of records received from a location and average murmuration size; and (2) the size and duration of murmuration events. Separate one-way ANOVA were used to determine any association between habitat and: (1) murmuration size; and (2) murmuration duration. To compare murmuration size and duration in the UK with non-UK sites, we used independent t-tests. To identify any patterns in murmuration size or duration with location across the UK, we correlated these variables against both latitude and longitude.

Temporal patterns in the size and duration of murmuration events in the 2014/15 data were analysed using curvilinear regression, with day as the independent variable (1 = 1 October 2014; 174 = 23^rd^ March 2015). The optimal model was selected based on R^2^. Linear regression was used to establish any pattern between day length and murmuration duration. As day length was averaged spatially over the UK (see above) this analysis was undertaken using data aggregated into weeks rather than using the raw daily data. This reduced the risk of days with atypically clustered data points (e.g. all records on an individual day coming, by chance, from the north of Scotland) biasing analysis.

To analyse the effect of temperature and predator presence/activity on murmuration size and murmuration duration, Regression with Empirical Variable Selection (REVS) was used [48]. This is superior to stepwise algorithms, which although highly intuitive, can be inconsistent, only test a small number of possible models, and can miss the optimal model because of the one-at-a-time nature of adding variables [49–52]. The alternative of all-subsets regression, whereby numerous models are created and compared using Akaike’s Information Criterion (AIC) [53], is more robust but the number of models generated increases exponentially with the numbers of predictors. The results can also be difficult to interpret when multiple (often very different) models are generated that have similar support, as shown by work by on bird-habitat associations [52]. REVS involves a series of n models being created (n = the number of predictors); the first containing the variable with most empirical support, the second containing that and the next most-supported, and so on. The resultant models are compared post-hoc using (AIC). This means: (1) the number of models needing comparison is lower (as n = the number of predictors rather than many times that number, as are typically generated with other approaches) and (2) all competing models have many variables in common (i.e. the “core” is the same; just minor differences in presence/absence of additional variables), which makes interpretation easier. REVS has been used in a range of ecological studies [54–55], including those using citizen science data [56].

Here, the REVS process was run four times, twice with murmuration size as the dependent variable and twice with murmuration duration as the dependent variable. The first analysis in each case included temperature, the *presence* of each predator species recorded (kestrel, peregrine, sparrowhawk, red kite, buzzard, harrier, owl) and the presence of avian species that could be mistaken for potential predators (corvids and gulls): n = 10 predictors. The second analysis in each case included temperature and the *activity* of each predator species (perching silent, perching calling, flying, or engaging with starling flock): n = 29 predictors (7 species * 4 behaviours = 28, plus temperature). It was necessary to run these analyses separately because presence and activity of each predator species were highly correlated (indeed, on a per-species basis, presence was the sum of all four activity data columns), which confounded orthogonality constraints [57]. Models were compared post-hoc using AIC based on ΔAIC < 2, while R^2^ was used to assess the biological significance of models. Although p values are arguably not important in AIC-driven analyses, they were given at model-level so overall statistical significance could also be assessed. For each dependent variable under consideration, three models were reported: (1) Minimum adequate model—the most parsimonious model (fewest predictors whilst still attaining ΔAIC < 2); (2) Optimal—the model that best balanced the number of variables and explanatory power (ΔAIC = 0); and (3) Maximum—the model that increased R^2^ to the maximum possible within the ΔAIC < 2 limit. Spatiotemporal autocorrelation was checked for each model using the Durbin-Watson test with the underlying data formatted so records were ordered by time within location to provide an appropriate data structure; in all cases the values were between 1.5 and 2.5 (a value of 2 signifies no autocorrelation and ± 0.5 is well within the accepted range given by Field [57]). There were no duplicate records for the same site on the same night and thus pseudoreplication was avoided.

### Ethics

It was not necessary to seek or obtain ethical clearance for this study since all work was solely based on non-invasive observation. No animals were handled, approached, or otherwise affected by the work undertaken.

## Results

In total, 3,211 records were submitted with the records split between years as detailed in Table 1. The end of the murmuration event was seen in 67.1% of cases. Based on data from 2014/15, when the exact date that the murmuration had been observed was provided, there was no relationship between the lag time from sighting and reporting a murmuration event (number of days: min = 0; max = 44; mean = 3) and its size or duration (Pearson correlation: r = 0.040, n = 553, p = 0.346 and r = 0.044, n = 553, p = 0.305, respectively).

**Table 1:** The number of records submitted and the numbers used in analyses after filtering.

Details	Year 1 (2014/15)	Year 2 (2015/16)	Total
Total number of records submitted	1,644	1,567	3,211
Number of non-UK records	68	123	191
Number of UK records after data cleaning (see Methods) that were accepted as murmurations (≥500 birds) but where the end of the murmuration may or may not have been witnessed.	1,293	1,134	2,427
Reduced dataset only containing UK records accepted as murmurations (≥500 birds), when the end of the murmuration was witnessed, and when all birds descended *en masse* to roost	553	513	1,066

The overall mean number of birds per murmuration was 30,082 ± 6,699 SEM and mean duration was 26 minutes (± 44 seconds SEM). There was a significant positive correlation between the size of murmuration and its duration (2014/14: r = 0.133, n = 577, p = 0.001; 2015/16 r = 0.127, n = 512, p = 0.004), but this relationship was extremely weak (R^2^ = 0.017 and 0.016, respectively).

### Spatial patterns

**Fig 1:**
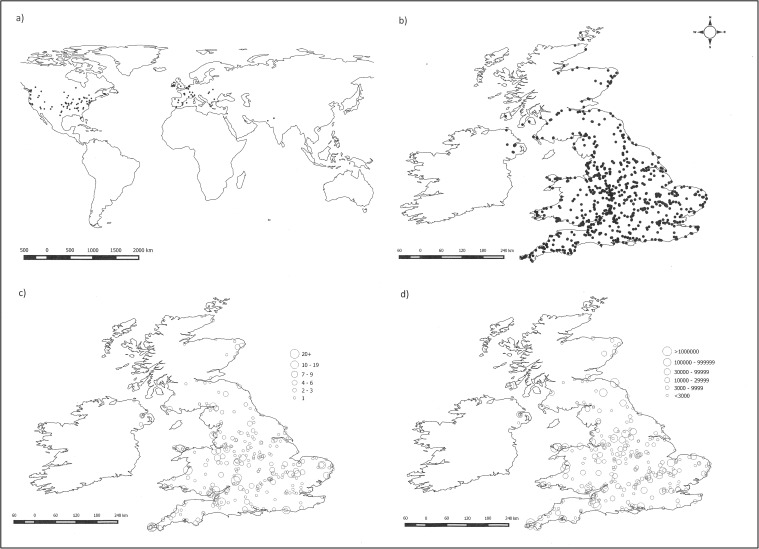
Reported starling murmurations: (a) = international distribution (; (b) UK basic distribution; (c) = UK distribution showing number of records; (d) = UK distribution showing mean size of murmuration. All base maps from Natural Earth (freely available in the Public Domain); all starling data from Starling Survey run by authors and freely available–see Data Availability Statement. Maps created using QGIS under CC BY.

The survey was envisioned as UK-based but 191 records (~6% of total) were submitted from outside the UK from 22 different countries. In total, 29 international records were not considered to be actual murmurations based on our data cleaning protocol (see Methods). The remaining records were from: USA (70), Canada (30), Netherlands (13), Eire (10), Italy (10), Spain (10), France (5), Belgium (3), Mexico (3), Bulgaria (2), Greece (2), Hungary (2), India (2), Australia (1), Germany (1), Jordan (1), Pakistan (1), Switzerland (1), and Ukraine (1); Fig 1A.

In terms of the UK, records came from as far south as Penzance in Cornwall 50.1190° N, 5.5370° W; as far north as Thurso in the Scottish Highlands, 58.5960° N, 3.5210° W; as far east as Lowestoft in Suffolk, 52.4800° N, 1.7500° E (the most easterly point of the UK); and as far west as Ballymoney, County Antrim, Northern Ireland 55.0710° N, 6.5080° W (Fig 1B). There were also records from offshore islands: Orkney, Skye and Arran (Scotland); Anglesey (Wales); and the Isle of Wight (England). There were some interesting patterns in the number of murmuration records from different sites (Fig 1C), with popular hotspots including Brighton and Aberystwyth piers (Southeast England and West Wales, respectively) and Gretna Green on the west coast near the English/Scottish border. There were also spatial patterns in the average size of murmuration events (Fig 1D), with large murmurations reported on the Somerset Levels in Southwest England, near Berwick-upon-Tweed on the east coast of the English/Scottish border, and Anglesey off the Welsh coast. There was no correlation between the average size of murmurations at a given location and the number of records received from that location (r = 0.044, n = 308, p = 0.443).

There was no significant difference in average murmuration size between the UK and either all non-UK records or the subset of non-UK murmurations that occurred outside the species’ native range in the USA/Canada (independent t-test: t = 0.604, d.f. = 545, p = 0.546 and t = 0.995, d.f. = 530, p = 0.320, respectively). However, UK murmurations lasted longer (mean = 26 minutes ± 44 seconds SEM) than all non-UK murmurations (mean = 18 minutes ± 2.5 minutes SEM) or the USA/Canada subset (mean = 16 minutes ± 3 minutes SEM). These differences were significant (UK vs. non-UK: t = -2.849, d.f. = 548, p = 0.005; UK vs. USA/Canada: t = -3.077, d.f. = 533, p = 0.002). These analyses were all based on data from 2015/16 when most international records were submitted and used only the reduced datasets (following criteria outlined above: ≥500 birds; end of murmuration seen). Within-UK patterns were analysed using 2014/15 data as exact location was only requested in the first survey. There was no significant relationship in murmuration size with either latitude (F_1,549_ = 2.510, p = 0.114) or longitude (F_1,549_ = 0.326, p = 0.568). There was a significant relationship between murmuration duration and latitude (F_1,549_ = 11.693, p = 0.001) with murmurations lasting longer further north but the amount of variance explained was low (r^2^ = 0.021). The was no relationship between duration and longitude (F_1,549_ = 3.114, p = 0.078).

### Habitat

**Fig 2:**
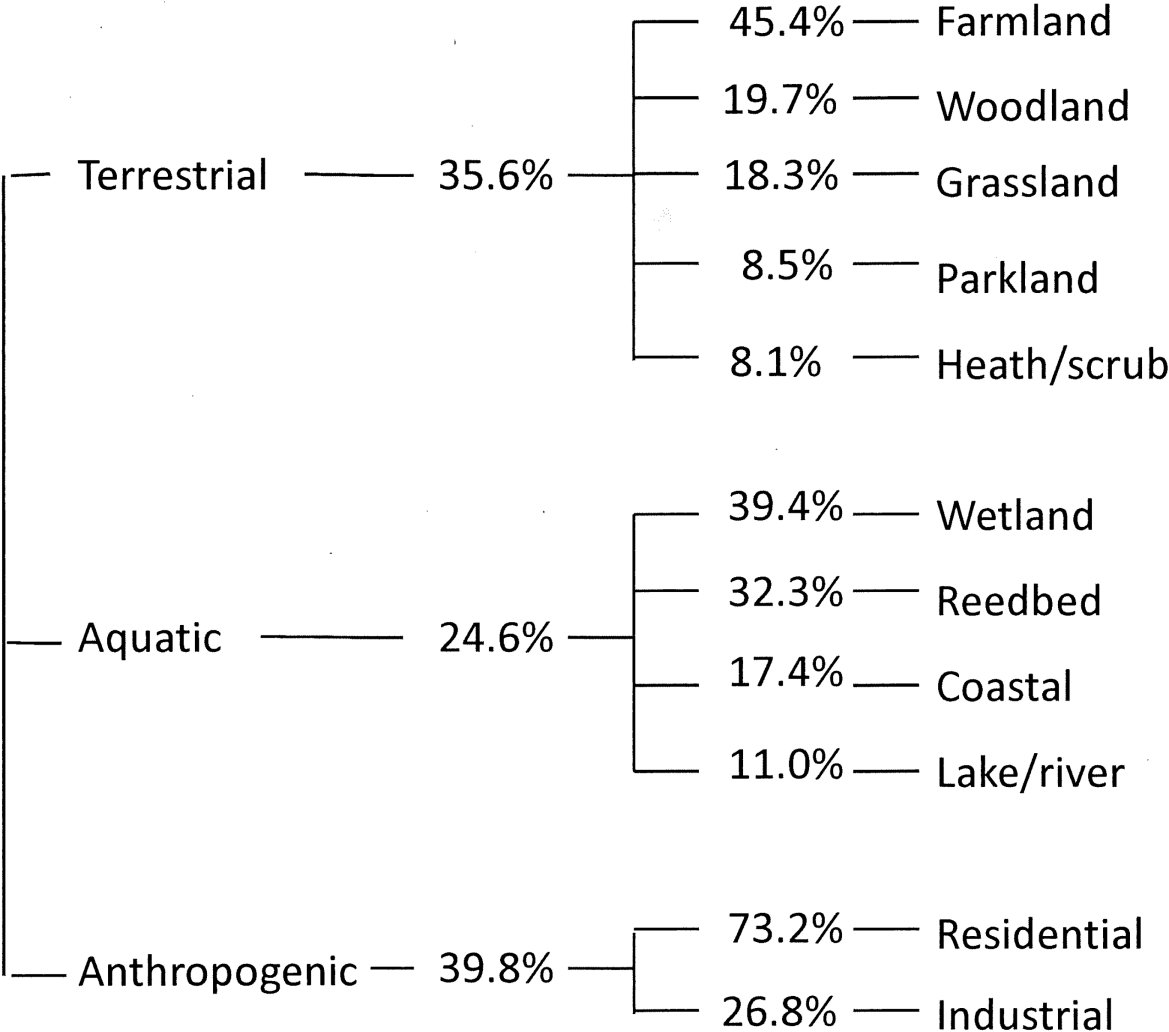
Number of murmurations associated with different landscapes (terrestrial, anthropogenic, aquatic) and different habitats with those landscapes based on data from survey year one (2014/15); n = 1,293.

In 2014/15, participants recorded habitat for each murmuration event. Of the UK murmurations, 61.2% occurred in rural areas, 19.2% in suburban and 19.6% in urban. The distribution of murmurations across habitat types is shown in Fig 2. Analysis on the subset of the data with ≥500 birds and the end of the murmuration was observed revealed no association between the habitat and either the size of the murmuration or its duration (ANOVA: F_11,645_ = 0.941, p = 0.500 and F_11,641_ = 1.217, p = 0.272, respectively). Roosting sites included elevated vegetation (especially coniferous trees and hedgerows), tall vegetation (especially when surrounded by water, as in the case of reedbeds or emergent marsh vegetation) and elevated manmade structures such as piers and building ledges.

### Temporal patterns

**Fig 3:**
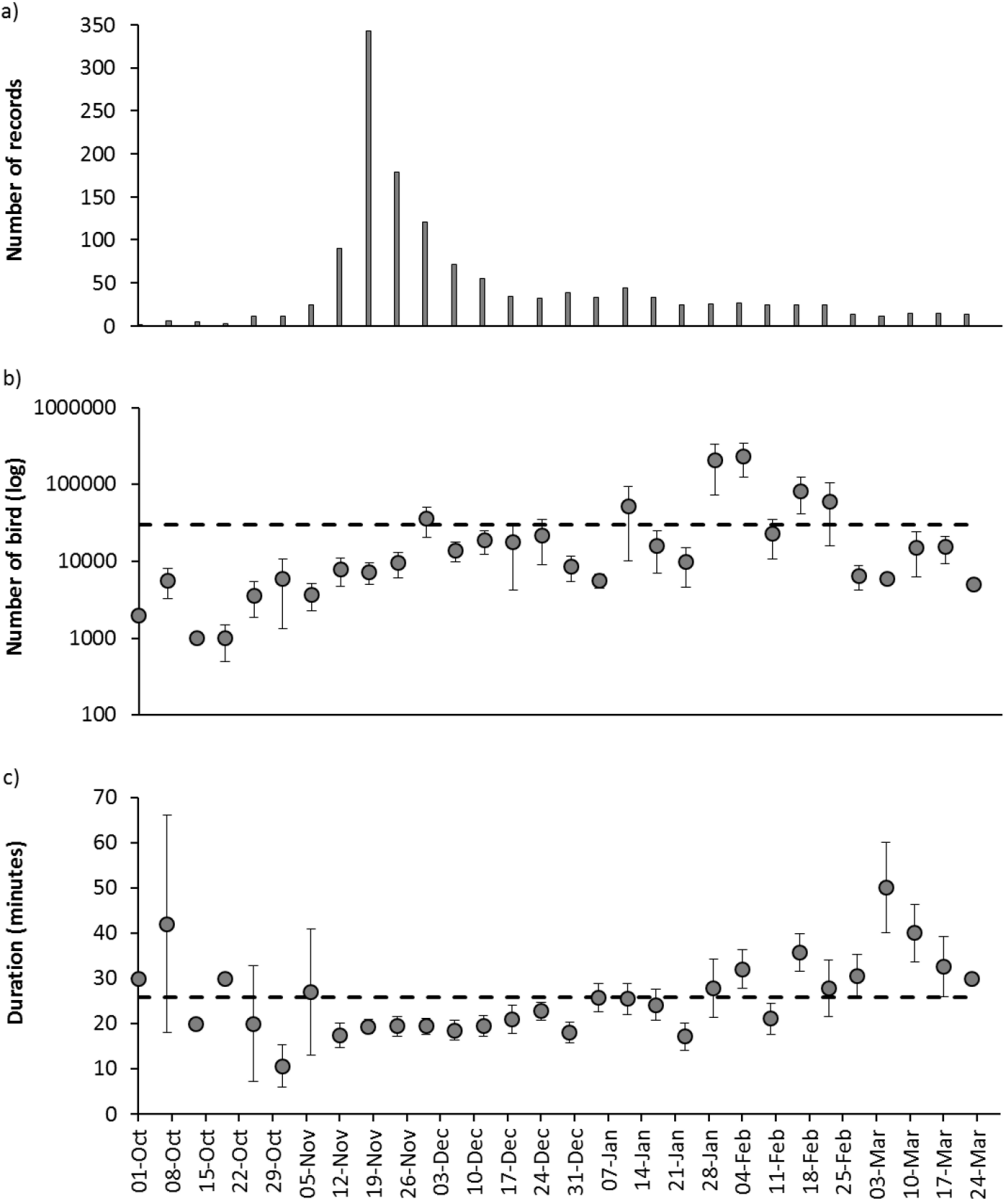
Temporal patterns in: (a) number of murmurations (n = 1,644); (b) mean number of birds per confirmed murmuration (n = 1,293); and (c) mean duration of murmuration only including records where the end of the murmuration event was recorded (n = 553). All data based on data from survey year one (2014/15); more details on sample sizes are given in Table 1. Dotted lines show annual means. Error bars show standard error.

Based on temporal data collected in 2014/15, there was a clear pattern in the number of records over time (Fig 3A), but this largely coincided with the main dates of publicity rather than any potential underlying seasonal pattern. There was a clear temporal trend in the mean size of murmurations, with the average number of birds increasing throughout the season until a peak in early February, after which size decreased again (Fig 3B). This was best explained by a quadratic curvilinear regression (F_2, 573_ = 4.671, p = 0.010; R^2^ = 0.127), which essentially described a negatively-skewed bell-shaped curve (y = 3.539*x*_1_^2^ + 1264*x*_1_−53688; Fig 3A).

**Fig 4:**
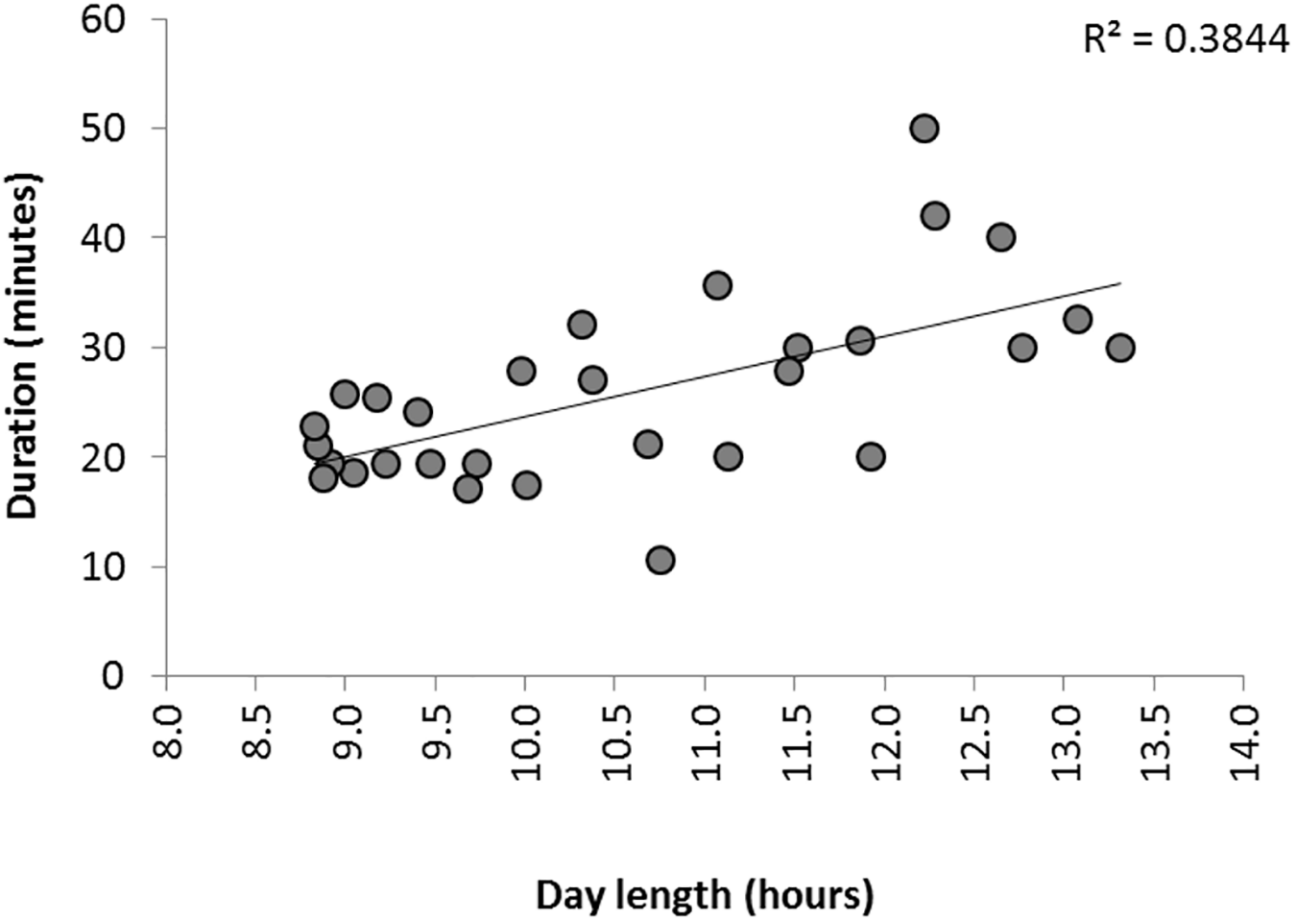
Relationship between murmuration duration (weekly mean duration in minutes) and day length in 2014/15 based on records where the end of the murmuration event was recorded (N = 553).

There was also a temporal trend in the duration of murmurations, which were longer at the beginning and end of the season and shorter in the middle (Fig 3C). This was best explained by a quadratic curvilinear regression (F_2,569_ = 21.877, P < 0.001, R^2^ = 0.267), which essentially described a shallow u-shaped distribution (y = 0.001*x*_1_2–0.065*x*_1_ + 19.915; Fig 3C). This pattern reflected seasonally variation in day length with murmurations being at their shortest around the winter solstice (i.e. in the middle of the study period). Reflecting this, there was a strong positive correlation between murmuration duration and day length (F_1,28_ = 17.488, P < 0.001, R^2^ = 0.384; Fig 4). It should, however, be noted that longer durations at the start/end of the season (>30 minutes) were based on fewer records and so the precision of the mean is reduced (see larger error bars in Fig 3C). As murmurations in early October were recorded only after the survey launched in mid-October (year 1) or early-November (year 2), recording bias might be partly responsible for early-season murmurations being above-average in duration by virtue of being more memorable. However, given that only 2–4 weeks elapsed between survey launch and the earliest accepted record of 1^st^ October, and that there was no relationship between sighting-to-reporting lag time and murmuration duration (see above), this is unlikely to be an important bias.

### Potential predators

**Fig 5:**
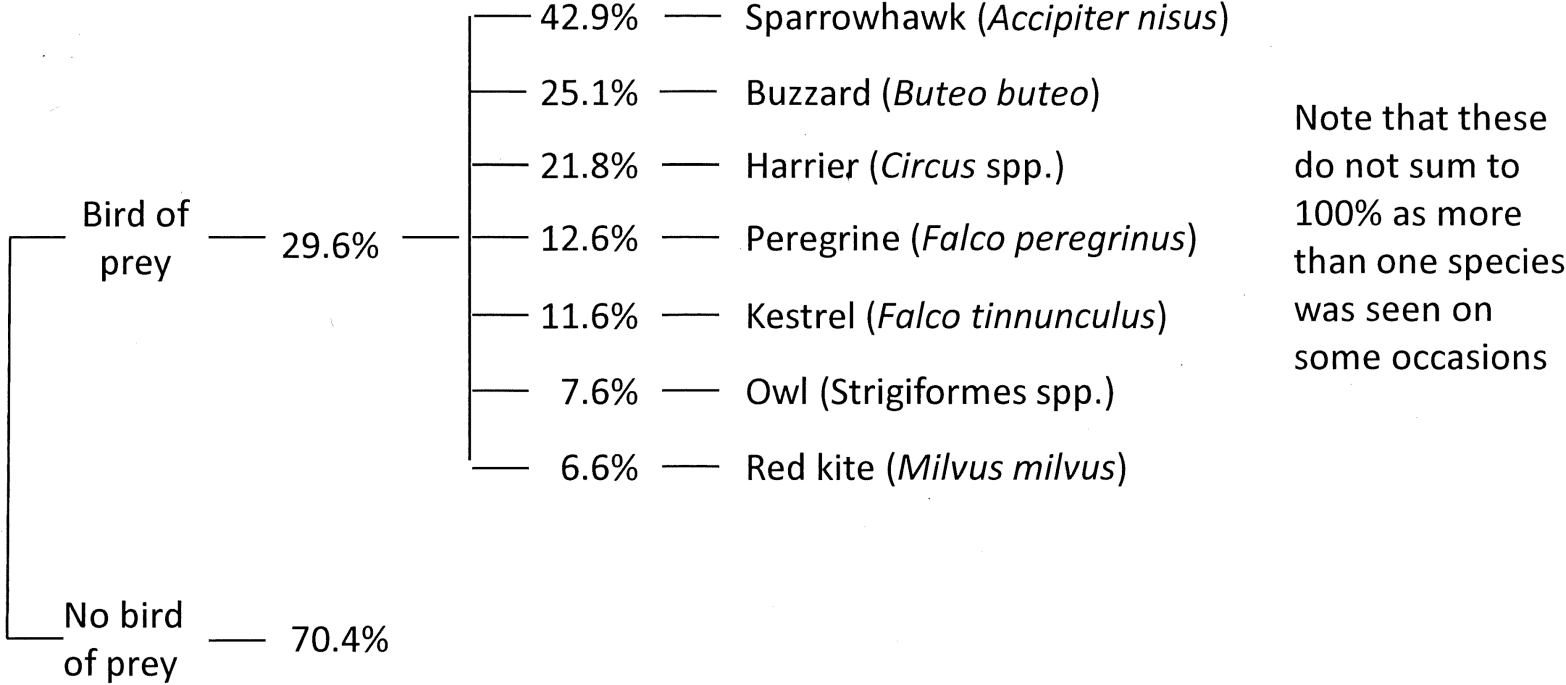
Number of murmurations associated with potential predators based on data from survey year two (2015/16); n = 1,134.

Birds of prey were recorded at 29.6% of murmurations. The most common species was sparrowhawk followed by buzzard, marsh harrier, hen harrier, and peregrine falcon (Fig 5). In addition, 15.8% of observers mentioned corvids and 17.6% mentioned gulls. These are not birds of prey, but were included because they might appear similar in silhouette and elicit a predator-avoidance response, especially given the low-light conditions at dusk.

### Explanatory models: Murmuration size and duration

The presence of potential predators was positively correlated with murmuration size. Harrier presence was added into the explanatory model first, followed by presence of buzzard, peregrine and owl. Adding sparrowhawk presence improved the final model, which explained 35% of variance in murmuration size and was highly significant (Table 2). All relationships were positive, such that murmurations were larger when potential predators were present (or, potentially, predators were more likely to be found at larger murmurations: see Discussion). Temperature and presence of non-predator species (corvids/gulls) were not significant. When presence of predators was replaced with their activity, similar models were created. Interestingly, although four activities were recorded for all predator species (perching silent, perching calling, flying near to the starling flock, and activity engaging with starlings by flying through the flock or making a predatory strike), it was only “flying” and “engaging” that ever met the model entry criteria. Harrier (engaging; flying) and peregrine (flying; engaging) were the four most important variables, followed by buzzard (engaging). All relationships were positive. The final model explained 40% of variance in murmuration size, which was higher than models using predator presence alone (Table 2). Again, temperature was not significant.

**Table 2:** Hierarchical regression models to explain murmuration size (number of birds) and murmuration duration (minutes) based on either temperature and predator presence or temperature and predator activity (perching silent, perching calling, flying, interacting with flock). In all cases, three models were created: (1) Minimum Adequate Model (MAM)–the most parsimonious model (i.e. the model that had fewest predictors whilst still attaining ΔAIC < 2); (2) Optimal—the model that best balanced the number of variables and explanatory power (i.e. ΔAIC = 0); and (3) Maximum—the model that increased adjusted R^2^ to the maximum possible within the ΔAIC < 2 limit. See methods for more details.

Dependent variable	Candidate independent variables	Model	Independent variables added (all relationships are + unless otherwise stated)	ΔAIC	Adj. R^2^	P
Murmuration size	Temperature; predator *presence*	MAM	Harrier, buzzard, peregrine	1.703	0.304	0.001
		Optimal	As MAM, plus owl	0.000	0.310	<0.001
		Maximum	As Optimal, plus sparrowhawk	1.430	0.349	<0.001
Murmuration size	Temperature; predator *activity*	MAM	As Optimal	-	-	-
		Optimal	Harrier (engaging), harrier (flying), peregrine (flying), peregrine (engaging)	0.000	0.385	<0.001
		Maximum	As Optimal, plus buzzard (engaging)	1.282	0.401	<0.001
Murmuration duration	Temperature; predator *presence*	MAM	Sparrowhawk, Temperature (-)	0.354	0.110	0.045
		Optimal	As MAM, plus harrier	0.000	0.118	0.033
		Maximum	As Optimal, plus buzzard, peregrine,	2.000	0.122	0.003
Murmuration duration	Temperature; predator *activity*	MAM	Sparrowhawk (engaging), sparrowhawk (flying), Temperature (-)	1.957	0.128	0.016
		Optimal	As MAM, plus, harrier (engaging), harrier (flying)	0.000	0.203	0.006
		Maximum	As Optimal, plus peregrine (engaging), buzzard (flying), kite (flying)	1.887	0.256	0.004

Both predators and temperature were significantly related to murmuration duration. The first model (using predator presence) showed that murmuration duration was significantly related to sparrowhawk presence (positive) and temperature (negative). Adding presence of harrier, buzzard and peregrine (all positive) improved the model but the variance explained was fairly low at 12%; presence of non-predatory corvids and gulls was not significant (Table 2). When the presence-only model was replaced by one that accounted for the behaviour of potential predators, the variance explained increased substantially. As with models of murmuration size, the only predator activity variables entered into the duration model were those that involved potential predators either flying near to the starling flock or actively engaging with it. The initial minimum model included sparrowhawk (engaging and flying; both positive) and temperature (negative). This model was improved by the addition of harrier (engaging and flying), peregrine (engaging), buzzard (flying), and kite (flying); all relationships were positive. The final model explained 26% of variance (Table 2). Analysis of temperature and duration alone (regression: F1,439 = 9.611, P = 0.002, R2 = 0.144) was weaker than the relationship between duration and day length (P < 0.001, R2 = 0.384; see above for full details).

### The end of the murmuration event

The frequency of birds descending *en masse* after murmurating was substantially and significantly higher, and the frequency of the murmuration dispersing and the birds flying away was lower, when a bird of prey was present compared to when a bird of prey was not present (chi square test for association: x^2^ = 33.600, d.f. = 2, P < 0.001). There was no difference in murmuration ending relative to temperature (one-way ANOVA _F2,487_ = 1.755, P = 0.174).

## Discussion

Using a citizen science approach, we gathered more than 3,000 records of starling murmuration behaviour from across the UK (and internationally) and covering two years. This is a far larger murmuration dataset both in terms of number of events and spatiotemporal scale than has previously been possible [13, 58]. Even after applying stringent filtering criteria, enforcing a minimum estimated size of 500 birds and requiring observers to see the end of the murmuration, there were >1,000 analysable murmuration observations.

Our analyses show that murmurations are not only widespread in terms of geographical scale, but also occur across a variety of habitats. This suggests that suitable roosting sites are widely distributed and are not confined to a few land-use types (unlike winter foraging, where starlings show a strong preference for permanent fields with abundant high-energy prey such as leatherjackets Tipulidae [59]). This implies that birds might travel some distance between feeding and roosting sites, an idea supported by individual birds regularly travelling ≥8 km between roost and feeding grounds [60] and exceptionally up to 50 km in favourable weather [61]. Individuals can switch roost (and thus murmuration) site to take advantage of overnight opportunities near their chosen feeding ground [60], which leads to turnover in the individuals involved in murmurations at specific sites on consecutive nights. Using citizen science could have led to geographical spread being under-recorded (since people are most likely to observe murmurations that are most accessible), but we found no relationship between the number of records from a location and murmuration size such that we can discount “honeypot murmurations” (i.e. specific murmurations becoming natural attractions) becoming over-represented.

Our data allowed empirical testing of competing hypotheses for the purpose of murmuration behaviour in starlings. The “warmer together”, or thermal, hypothesis predicts that murmuration size and duration will be inversely correlated with temperature since the need for roost advertising should be greater in colder conditions. However, although low temperatures have previously been found to be important in promoting flocking behaviour [9, 35], we found only weak support for this hypothesis in the specific case of starling murmurations. Temperature was not a significant predictor of murmuration size although it was significantly negatively related to duration (colder evenings had longer murmurations). Duration was, however, more strongly related to day length than temperature (R^2^ = 0.384 versus R^2^ = 0.144), with longer murmurations tending to occur at the beginning and end of the season and shorter murmurations occurring in the darkest (and usually coldest) winter weeks.

Predator presence has been shown to affect shapes and waves within murmurations [13–14, 44]. Our data have allowed, for the first time, robust analysis of multiple predator species on murmuration size and duration. Our analyses support predators being important in murmuration behaviour (the “safer together” hypotheses) but determining cause and effect is problematic. Predators were present at ~30% of murmurations and their presence was significantly positively associated with both size and duration. However, larger and longer murmurations are undoubtedly far more visible to birds of prey than smaller, shorter, murmurations. Thus, while the dilution effect means individual predation risk is lower in a larger group (as found for other taxa, including marine insects, European minnows, and sand fiddler crabs [30, 33–34]), being part of a larger group may actually increase the per-group strike rate, as found in whirligig beetles Gyrinidae [62]. The murmuration-predator relationship could, therefore, be as much (potentially more) driven by murmurations attracting predators–and larger murmurations being more attractive–than by predators causing starlings to murmurate. Interestingly, though, it was only birds of prey, rather than crows or gulls, that were linked to murmuration size and duration, suggesting that murmurating starlings might be able to distinguish predators from visually similar species (although note that the sample sizes were much smaller for crows and gulls, which might have had an effect). Additional evidence for the causal role of predator defence in murmuration behaviour is provided by the observation that only when predators were flying near, or actively engaging with, the murmuration (i.e. when they could pose a direct and immediate predation risk) did they affect murmuration size/duration. Our analyses also show that predator presence was related to starling behaviour at the end of murmuration: it was more likely that birds would descend *en masse* if a bird of prey was present. In contrast, starlings were more likely to disperse in the absence of predators. This terminal behaviour is, in many ways, more revealing than size/duration correlations when considering the role of predators: if murmurations were not in some way related to predator defence then terminal behaviour is unlikely to be correlated with predator presence.

A citizen science approach enabled a substantial dataset to be collected over a wider spatiotemporal scale than would have otherwise been possible. However, this approach is not without limitation. In addition to the location of records potentially reflecting the location of populations rather than, or in addition to, the focal phenomenon (see above), there is a risk of recording error among citizen scientists. Here, untrained observers were asked to give accurate estimates of bird numbers and, in common with much citizen science research, there was no way to validate the data. Inter-observer variation is likely; moreover because larger displays are likely to be harder to estimate accurately, recording error might not be independent of display size leading to heteroscedasticity. Any such patterns in the data would not be identifiable. One way to address this in future studies would be to ask respondents to submit a photograph or–better in the case of behaviour–video footage so data could be extracted by a single trained expert rather than a large number of untrained members of the public. This would increase researcher time but, where this is possible, would help improve data quality and consistency. This approach has been used previously to confirm species identification in citizen science surveys [63]. Where this is not possible, use of an online quiz to assess observers’ skill so that this could be used to weight their data post-submission (as per [64]) might be helpful.

Despite the limitations outlined above, our findings suggest that the collective behaviour observed in starling murmurations is primarily an anti-predator adaptation rather than a way of attracting larger numbers of individuals to a roost for warmth. Suitable roosting sites attract large numbers of birds who would be vulnerable flying to the roost individually. Murmurating above the roosting site provides multiple advantages in terms of the dilution effect [29], increased vigilance leading to the detection effect [37–39] and predator confusion [41]. This model of murmuration relies on having a critical mass of birds arriving at more-or-less the same time to initiate the murmuration and further study of the behaviour of starlings at the start of the murmuration (and indeed, just before the start of the murmuration) would be valuable in unravelling how this behaviour develops from a relatively few number of individuals into a spectacular collective behaviour comprising potentially tens of thousands of individuals.
